# Oligonucleotides and microRNAs Targeting Telomerase Subunits in Cancer Therapy

**DOI:** 10.3390/cancers12092337

**Published:** 2020-08-19

**Authors:** Adam Eckburg, Joshua Dein, Joseph Berei, Zachary Schrank, Neelu Puri

**Affiliations:** Department of Biomedical Sciences, University of Illinois College of Medicine at Rockford, Rockford, IL 61107, USA; aeckbur2@uic.edu (A.E.); jdein2@uic.edu (J.D.); jberei2@uic.edu (J.B.); schrank.zachary@gmail.com (Z.S.)

**Keywords:** anti-cancer therapy, oligonucleotides, microRNAs, antisense oligonucleotides, telomerase, hTERT, T-oligo, 6-thio-dG, G-quadruplex

## Abstract

Telomerase provides cancer cells with replicative immortality, and its overexpression serves as a near-universal marker of cancer. Anti-cancer therapeutics targeting telomerase have garnered interest as possible alternatives to chemotherapy and radiotherapy. Oligonucleotide-based therapies that inhibit telomerase through direct or indirect modulation of its subunits, human telomerase reverse transcriptase (hTERT) and human telomerase RNA gene (hTERC), are a unique and diverse subclass of telomerase inhibitors which hold clinical promise. MicroRNAs that play a role in the upregulation or downregulation of hTERT and respective progression or attenuation of cancer development have been effectively targeted to reduce telomerase activity in various cancer types. Tumor suppressor miRNAs, such as miRNA-512-5p, miRNA-138, and miRNA-128, and oncogenic miRNAs, such as miRNA-19b, miRNA-346, and miRNA-21, have displayed preclinical promise as potential hTERT-based therapeutic targets. Antisense oligonucleotides like GRN163L and T-oligos have also been shown to uniquely target the telomerase subunits and have become popular in the design of novel cancer therapies. Finally, studies suggest that G-quadruplex stabilizers, such as Telomestatin, preserve telomeric oligonucleotide architecture, thus inhibiting hTERC binding to the telomere. This review aims to provide an adept understanding of the conceptual foundation and current state of therapeutics utilizing oligonucleotides to target the telomerase subunits, including the advantages and drawbacks of each of these approaches.

## 1. Introduction

The terminal portions of human chromosomes are capped by nucleoprotein structures called telomeres [1,2,3,4]. Telomeres maintain genomic stability in normal somatic cells and protect against chromosomal degradation [5,6]. Telomeres are progressively shortened by each round of cell division, and, after approximately 50 divisions, they reach a critically short length that induces DNA damage responses leading to cellular senescence or apoptosis [7,8,9]. Telomerase is an enzymatic ribonucleoprotein complex that maintains telomere length by adding single-stranded TTAGGG stretches to the 3′ end of the chromosome [6,8,10,11,12]. Human telomerase is composed of two subunits, a reverse transcriptase component called human telomerase reverse transcriptase (hTERT) and a template RNA component called human telomerase RNA gene (hTERC) [11,12,13]. These two subunits adopt a well-defined tertiary structure in the catalytic core of the telomerase complex and exhibit limited protein-RNA interaction [12]. Telomerase activity is relatively quiescent in most normal tissue; however, studies have found it to be significantly increased in up to 90% of malignancies [8,14,15,16]. The activation of telomerase allows cells to circumvent senescence and continue to divide while accumulating oncogenic mutations that can ultimately lead to cancer [17,18]. Cancer therapeutics specifically targeting telomerase-positive cells have garnered interest as a possible alternative to traditional chemotherapy and radiotherapy methods, which are toxic to both cancerous and normal cells [8,15]. The use of oligonucleotides and microRNAs (miRNAs) that inhibit the function of hTERC and hTERT is a further specified subclass of telomerase-centered therapies that has shown promise in treatment for a number of human malignancies [19,20,21,22]. This comprehensive review will elaborate on the current state and underlying concepts of cancer therapeutics aimed at reducing telomerase activity by targeting telomerase subunits with oligonucleotide-based approaches.

miRNAs are endogenous oligonucleotides that play an important role in post-transcriptional gene regulation. miRNAs often target genes involved in cancer-related processes, such as the expression of hTERT, the catalytic subunit of telomerase [23]. Most tumor suppressor miRNAs downregulate hTERT through direct interaction with the 3′-untranslated region (3′-UTR) of hTERT mRNA, while oncogenic miRNAs indirectly upregulate hTERT activity through inhibition of genes involved in the suppression of hTERT [22]. The therapeutic manipulation of miRNA-mediated hTERT expression to treat human malignancies largely remains in preclinical stages; however, the array of potential miRNA targets explored in this review demonstrates the clinical promise of this approach [15]. Antisense oligonucleotides (AS-ODNs) like GRN163L (Imetelstat) and T-oligos have also been utilized to target telomerase activity through inhibitory interactions with hTERC and hTERT, respectively, and GRN163L has progressed into clinical trials [15,19,21,24,25,26,27,28]. Furthermore, telomeric G-quadruplex (G4) secondary structures formed by sequences rich in guanine serve as nucleotide sites intrinsic to telomere architecture that can also be targeted to modulate the action and expression of the telomerase subunits [25,29,30,31]. The therapeutic inhibition of telomerase through the activity of the aforementioned oligonucleotide targets has been thoroughly explored in a number of cancer types [28,32]. A comprehensive understanding of these anti-cancer approaches is crucial for the development and improvement of new telomere-based cancer therapies that could serve as possible alternatives to traditional chemotherapy-based treatment modalities. 

## 2. MicroRNAs 

**Figure 1:**
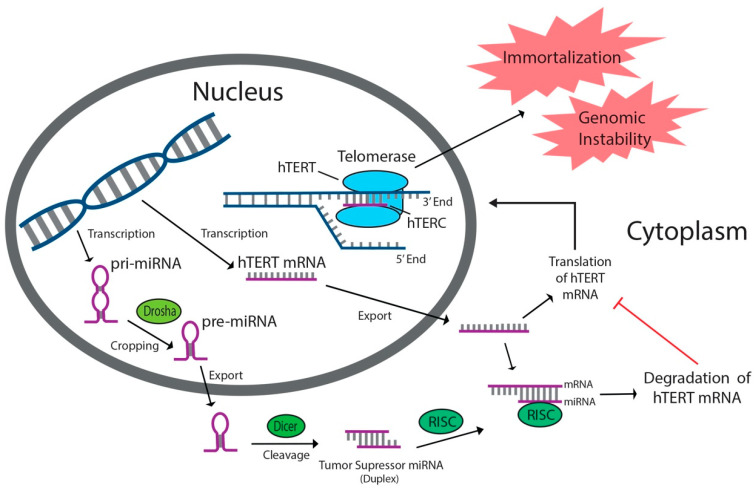
Tumor suppressor miRNAs targeting human telomerase reverse transcriptase (hTERT) inhibit telomerase activity by binding to hTERT mRNA and preventing productive translation of the reverse transcriptase component of telomerase. miRNAs are transcribed in the nucleus, cleaved by Drosha and Dicer, then associated with RNA-induced silencing complex (RISC) in the cytoplasm. Tumor suppressor miRNAs prevent angiogenetic characteristics associated with increased telomerase activity in cancers.

miRNAs comprise a non-coding class of RNA in eukaryotes that stretch 18–24 nucleotides in length and function as endogenous post-transcriptional regulators of gene expression [8,33,34,35]. Primary miRNAs (pri-miRNAs) are transcribed and cleaved in the nucleus by RNAse III Drosha to form hairpin-shaped pre-miRNAs (Figure 1) [36]. In the cytoplasm, these structures are cleaved by RNAse III Dicer to form mature miRNAs, which then associate with the RNA-induced silencing complex (RISC) (Figure 1) [34,37]. Once complexed to RISC, miRNA base pairs with a complementary sequence on its target messenger-RNA site (mRNA), typically within the 3′-UTR, and induces its degradation by RISC or prevents productive translation of the mRNA, thus effectively silencing the associated gene (Figure 1) [8,33,34,35,37,38]. More than 50% of gene sequences encoding miRNAs exist in regions associated with cancer and/or increased mutability [22]. As a result, miRNAs have been the subject of recent clinical attention due to their involvement in driving cancer progression or in silencing genes associated with cancer progression [35]. Oncogenic miRNAs facilitate cancer growth, while tumor suppressor miRNAs inhibit proliferation [35,39]. Many tumor suppressor and oncogenic miRNAs impact cancer development by directly or indirectly targeting hTERT, the reverse transcriptase component of telomerase, causing it to be downregulated or upregulated, respectively. Increased hTERT expression leads to increased telomerase activity and angiogenic characteristics, while hTERT downregulation limits telomerase activity and causes antiproliferative effects. A vast amount of therapeutic potential lies in the use of miRNA-induced gene modulation to regulate hTERT expression and prevent the overexpression of telomerase, which is a common characteristic of human cancers [35]. 

Some of the most well-studied hTERT-targeting tumor suppressor miRNAs include miRNA-1182, miRNA-133a, miRNA-342, miRNA-491, miRNA-541, miRNA-512-5p, miRNA-138, and miRNA-128 (Table 1) [15,20]. miRNA-1182 has been shown to reduce gastric cancer cell proliferation and migration by targeting the open reading frame-1 (ORF-1) of hTERT [40]. Furthermore, an inverse correlation between miRNA-1182 expression and hTERT protein levels in gastric cancer cells suggested that miRNA-1182 could be utilized as a biomarker and potential treatment for this form of cancer [40]. Other studies suggested that miRNA-1182 overexpression inhibited bladder cancer cell proliferation, colony formation, and invasion by binding to the 3′-UTR of hTERT transcripts [41]. miRNA-1182 has also been shown to slow metastasis in ovarian cancer cells through hTERT inhibition and thus holds promise as a potential therapeutic target for this form of cancer [42]. In HeLa adenocarcinoma cells, miRNA-133a was shown to bind directly to the 3′-UTR of hTERT mRNA, demonstrated through inhibition of TERT 3′-UTR-driven reporter activity [20]. In the same study, miRNA-342, miRNA-491, and miRNA-541 were each shown to bind the 3′-UTR of hTERT as well [20]. Combining all three miRNAs yielded increasing inhibitory effects, suggesting each miRNA binds to a unique region of the 3′-UTR on hTERT mRNA [20]. miRNA-512-5p is another tumor suppressor miRNA that has been shown to post-transcriptionally regulate hTERT through a unique 3′-UTR binding site on hTERT mRNA [43]. In head and neck squamous cell carcinoma (HNSCC) cells, miRNA-512-5p inhibited cell growth by decreasing telomerase activity, shortening telomere length, and disrupting telomere-binding proteins [43]. Tumor suppressor miRNA-138 has also been associated with antiproliferative effects and inhibition of tumor growth as a result of direct targeting of hTERT [44,45]. In human anaplastic thyroid carcinoma cells, miRNA-138 downregulation was associated with hTERT overexpression [15,45]. In cervical cancer cells, overexpression of miRNA-138 led to inhibition of cell proliferation, migration, and invasion, as well as induction of apoptosis, caused by binding of miRNA-138 to the 3′-UTR of hTERT mRNA [44]. Furthermore, upregulation of miRNA-138 proved to be more effective than hTERT knockdown in potentiating Apigenin-induced apoptosis, a process caused by the release and activation of caspases, in malignant neuroblastoma cells both in vivo and in vitro [46,47]. This suggests that miRNA-138 upregulation may serve as a promising alternate pathway to control tumor growth [47]. miRNA-128 is another unique miRNA that can act as both an oncogenic miRNA and a tumor suppressor miRNA depending on the tumor type [33,48,49]. miRNA-128 has been shown to interact with the coding sequence of hTERT mRNA in HeLa and teratoma cells [48]. In HeLa cells, overexpression of miRNA-128 led to reduced levels of hTERT mRNA and TERT protein, as well as reduced cell proliferation [48]. On the other hand, downregulation of miRNA-128 has been associated with metastasis and development of a number of malignancies, including glioma, prostate, head and neck, lung, and colorectal cancers [33,48]. 

Oncogenic miRNAs that directly or indirectly upregulate hTERT expression and drive cancer metastasis and aggression through increased telomerase activity can also be targeted as a therapeutic approach. Some of the most well-studied oncogenic miRNAs in this category include miRNA-19b, miRNA-346, and miRNA-21 (Table 1) [8,51]. Studies suggest that miRNA-19b expression indirectly upregulates hTERT expression through inhibition of a novel hTERT suppressor gene, paired-like homeodomain1 (PITX1) [51]. PITX1 is a negative regulator in the RAS signaling pathway that binds directly to the hTERT promoter region, repressing hTERT transcription and telomerase activity, as well as preventing cellular proliferation [51]. miRNA-19b binds to a complementary sequence in the 3′-UTR of PITX1 mRNA, repressing PITX1 translation and activating hTERT expression [51]. Overexpression of miRNA-19b led to 1.5- to 1.7-fold increases in telomerase activity in melanoma cells and has been associated with oncogenesis in lung cancer, melanoma, breast cancer, and osteosarcoma [50,51]. In cervical cancer cells, miRNA-346 has been shown to mediate hTERT upregulation through a competitive process with miRNA-138, which binds to the same region of the 3′-UTR of hTERT mRNA as miRNA-346 but promotes an opposite effect on hTERT expression [55]. The binding of miRNA-346 to the 3′-UTR induces the binding of G-rich RNA sequence binding factor 1 (GRSF1) to the miRNA-346 CCGCAU sequence [55]. This interaction forms a “bulge loop” that recruits hTERT mRNA to ribosomes for translation [55]. By way of this translation process independent of argonaute RISC catalytic component 2 (AGO2), miRNA-346 enhances proliferation in cervical cancer cells [55]. Conversely, when miRNA-138 binds, it mediates the suppression of hTERT translation in an AGO2-dependent manner [55]. Studies suggest that miRNA-21 regulates hTERT expression through several indirect pathways. miRNA-21 impacts telomerase activity through downregulation of phosphate and tensin homolog deleted on chromosome 10 (PTEN), a tumor suppressor gene tied to tumor progression through activation of the PI3K/AKT pathway [54]. In hypertrophic scar fibroblasts, transfection with a miRNA-21 mimic led to increased hTERT mRNA and protein levels as well as increased PI3K/AKT signaling [54]. Additionally, the introduction of PTEN cDNA inhibited the effects of miRNA-21 overexpression [54]. In malignant melanoma cells, miRNA-21 was found to be associated with hTERT upregulation, resulting in increased invasiveness, oxidative stress, genomic instability, and cell proliferation, as well as evasion of apoptosis [52]. Furthermore, in colorectal cancer, a 3.4-fold increase in miRNA-21 expression was correlated with a 2-fold increase in hTERT expression [53]. Studies have also shown that miRNA-21 enhances carcinogenesis through STAT3, a transcription factor that plays a key role in Th17 helper T-cell differentiation [15]. When miRNA-21 was knocked down in murine glioblastoma xenografts, reduced expression of both hTERT and STAT3 was observed, as well as slowed tumor growth [15].

A number of different therapeutic approaches targeting miRNAs to reduce hTERT expression and telomerase activity have been investigated. The administration of hTERT-targeting tumor suppressor miRNAs to act as telomerase inhibitors in combination with chemotherapy drugs has been explored in numerous clinical applications and shown to be more effective than use of chemotherapy drugs alone [56]. The use of anti-miRNA oligonucleotides (AMOs), which are synthetic sequences antisense to a specific miRNA, to reduce the activity of hTERT-targeting oncogenic miRNAs, has also garnered significant interest [57]. Treatment with anti-miRNA-21 oligonucleotides successfully reduced STAT3 and hTERT levels in cancer cells, while administration of anti-miRNA-19b to melanoma cells reduced hTERT mRNA expression and increased PITX1 mRNA expression [51,58]. Studies have shown that unique subsets, or “families,” of miRNAs tend to be upregulated together in cancer [15]. miRNA sponges are stretches of DNA with artificial miRNA binding sites incorporated into the 3′-UTR of a gene that are utilized to simultaneously downregulate a group of hTERT-targeting miRNAs [59]. A recent study in bladder cancer cells showed that synthetic miRNA sponges driven by a mutant hTERT promoter were successful in downregulating four oncogenic miRNAs, thus inhibiting cell growth, decreasing motility, and inducing apoptosis in cancer cells but not in normal cells [59]. Studies have investigated the use of small RNA-specific ligands targeting the narrow groove of specific pre-miRNAs to inhibit the formation of mature oncogenic miRNAs [60]. Finally, the use of CRISPR/Cas9 to knockdown genes encoding oncogenic miRNAs, most notably miRNA-21, has also been explored extensively and was shown to reduce the expression of oncogenic miRNAs by up to 96% [61,62].

A wide range of techniques that utilize tumor suppressor and oncogenic miRNAs to regulate hTERT expression and limit telomerase activity have been explored; however, these therapies remain largely in preclinical stages, likely due to inherent issues associated with telomerase inhibition. It often takes several weeks for telomerase inhibition to cause critical telomere shortening and anti-cancer effects, a long timeframe which compromises the efficacy of these therapies [63]. Furthermore, studies have suggested that critical telomere shortening may lead to deleterious side effects such as cell crisis, genomic instability, and cancer progression [64]. Previous research also suggested that telomerase may re-localize under certain stress conditions and participate in responses that remain poorly characterized [64]. The long-term effects of telomerase inhibition are also not well-understood because the specific action of telomerase after therapeutic targeting has not been fully characterized. Before miRNA therapies targeting hTERT can be broadly implemented as a clinical approach, an added depth of understanding and clarification regarding the exact mechanism of telomerase inhibition is required. Another issue limiting the applicability and effectiveness of these therapies is the tissue-specific expression patterns of different miRNAs [65]. Depending on the tissue type, some miRNAs can act as either a tumor suppressor or an oncogenic miRNA [48,65,66]. Future studies should take these limitations into account when designing miRNA-based therapies targeting telomerase for specific forms of cancer. Some miRNA-based therapies have progressed to clinical trials; however, none mechanistically target hTERT. Inhibition of miRNA-122 for treatment of hepatitis C has progressed to Phase II clinical trials [15]. Additionally, MRG-106 oligonucleotides used as antisense inhibitors to miRNA-155 for treatment of cutaneous T-cell lymphoma has moved into Phase I trials [15]. The clinical progression of these therapies demonstrates the promise of miRNA-based treatment approaches. The design of hTERT-targeting miRNAs that overcome the limitations of telomerase inhibition may hold significant clinical promise. Furthermore, due to the tissue specificity of miRNAs, the use of miRNAs as a blood and tumor biomarker in prostate, lung, and breast cancers has been thoroughly investigated [67,68,69]. Since hTERT upregulation also serves as a meaningful biomarker for several cancers, the diagnostic and prognostic utilization of hTERT-targeting oncogenic or tumor suppressor miRNAs is an approach with significant therapeutic potential [70].

## 3. Oligonucleotides

Oligonucleotides are molecules composed of short segments of DNA or RNA that have been used for a variety of purposes in the fields of medicine and biomedical research. Because of their ability to bind to complementary sequences of DNA or RNA, oligonucleotides have been employed as simple nucleic acid probes using a number of basic science laboratory techniques, such as polymerase chain reaction (PCR) and DNA sequencing, as well as in the specific targeting and regulation of the expression and function of a wide range of proteins [71,72]. Due to their diverse functionality, oligonucleotides have been at the center of many research studies in numerous disease models [73]. They have been particularly useful in studying cancer biology, as their ability to bind and regulate specific nucleotide sequences allows for the study of both the etiology and treatment of many different cancers [15,19,21]. Specific oligonucleotide molecules such as miRNAs and AS-ODNs, including T-oligos, have been used to target distinct complementary DNA and RNA sequences in order to modulate both aberrant protein levels and enzyme activities implicated in various cancers [8,15,74]. 6-Thio-2′-Deoxyguanosine (6-Thio-dG) is an effective nucleoside analog and telomerase substrate which brings about several anti-cancer effects through incorporation into DNA [8,15].

Much progress has been made in the use of oligonucleotides to target molecular events leading to the upregulation of telomerase [23,75]. As previously mentioned, hTERT is the catalytic component of telomerase that is essential for the replication of chromosomal telomeres, a process that confers cells with perpetual replicative potential [23,75,76]. Oligonucleotides can be tailored to target hTERT at specific steps in its synthesis and activity, including at the hTERT promoter, hTERT mRNA, or in its protein form [15,77]. In order for hTERT to successfully perform its catalytic function, it must be able to properly interact with telomere-related proteins in the shelterin complex and bind to the telomeric nucleotide sequence using hTERC. Regulation of the hTERT protein itself has been accomplished through oligonucleotide-based interference of hTERT at a number of different points [8,15].

AS-ODNs have shown promise in both preclinical and clinical trials as a potential treatment for various cancers. A number of preclinical studies used AS-ODNs to target the hTERT initiator sequence, pre-mRNA, or transcribed mRNA, which significantly decreased hTERT activity and increased growth inhibition in human hepatoma, prostate adenocarcinoma, bladder cancer, and hepatic lymphoma [24,78,79,80]. In particular, Folini et al. found that antisense oligonucleotide-mediated inhibition of hTERT, but not hTERC, inhibited cell growth and induced apoptosis in human prostate cancer cells, implying that telomerase can retain its role in telomere replication even in the absence of a properly functioning RNA template. Another study found that the simultaneous in vitro targeting of both hTERT and hTERC with AS-ODNS resulted in the synergistic inhibition of growth in human colon cancer cells [81]. The results of these early preclinical studies showed that hTERT could be successfully targeted at a number of regulatory points involved in its cellular production [81]. They also demonstrated the wide applicability of specifically engineered oligonucleotides to target cells with abnormal hTERT activity [81]. Additionally, various preclinical and clinical studies have revealed the synergistic anti-cancer effects of AS-ODNs with other cancer drugs on the inhibition of abnormal cancer cell growth [82,83,84].

Although AS-ODNs can be used to target the production of hTERT, one of the best-studied AS-ODNs GRN163L (Imetelstat) acts as a competitive inhibitor of telomerase activity. GRN163L is a 13-merthiophosphoramidate deoxyribo-oligonucleotide that is a complement and antagonist to the hTERT-associated hTERC RNA template sequence. By binding to hTERC, Imetelstat prevents hTERT-mediated telomere elongation, leading to progressive telomere attrition and eventual cell death [8]. Imetelstat preferentially targets cancer cells due to their intrinsically high level of telomerase activity and has been tested in a number of preclinical and clinical trials conducted to assess its effect on different types of cancers, with varying levels of success [82,84,85].

In preclinical studies using both in vitro human cells and in vivo murine models, Imetelstat has shown great promise in its ability to decrease the growth of many cancers, as well as downregulate hTERT activity. Imetelstat alone significantly decreased telomere length through hTERT inhibition and increased cell death of human myeloma and pancreatic cancer cells in an in vitro model [63,86]. Xenograft murine models showed similar results for both myeloma and malignant rhabdoid tumor cells, demonstrating telomere shortening, decreased growth, and increased cell death [86,87]. Additionally, Imetelstat has exhibited anti-metastatic properties in mouse models of lung cancer [88,89]. Imetelstat has been tested in numerous preclinical trials involving other types of cancers, both by itself and in combination with other anti-cancer therapeutics, with similar patterns of inhibition of telomerase and decreasing cell viability being observed [15,28]. Despite the apparent effectiveness of Imetelstat in preclinical studies, many preclinical trials using it have severe limitations which prevent its applicability to human treatments. Some of these issues include significant differences observed between in vitro and in vivo models for human cells as well as apparent variations between telomere structure and maintenance in mice compared to humans [15].

Despite its success in preclinical trials, Imetelstat has not had as much success in clinical trials due to its severe hematological side effects, including thrombocytopenia and myelosuppression, and as a result has not been approved by the U.S. Food and Drug Administration (FDA) [1]. It has been involved in numerous Phase I/Phase II clinical trials that have been completed as well as some Phase II/Phase III trials that are still ongoing [15,28,90]. These studies have shown mixed results, with some reporting no significant increase in long-term patient survival and others reporting no significant reduction in tumor size despite a 95% reduction in telomerase activity [91,92].

Preclinical and clinical trials have tested the drug both alone and in combination with other anti-cancer drugs in the treatment of various types of cancers [1]. The most promising results with Imetelstat have been obtained when it is used alongside other chemotherapeutic agents, such as 3-aminobenzamide (3AB) and trastuzumab, or conventional radiation therapy, possibly through the increased sensitization of cancer cells to these other treatments [15,93,94]. Future studies on Imetelstat should investigate the synergistic targeting of hTERT by Imetelstat in combination with other drugs, such as small molecule inhibitors, that target other sources of cancer aggression while simultaneously minimizing unfavorable toxicity. Both preclinical and clinical trials are currently ongoing in these areas [15,28].

**Figure 2:**
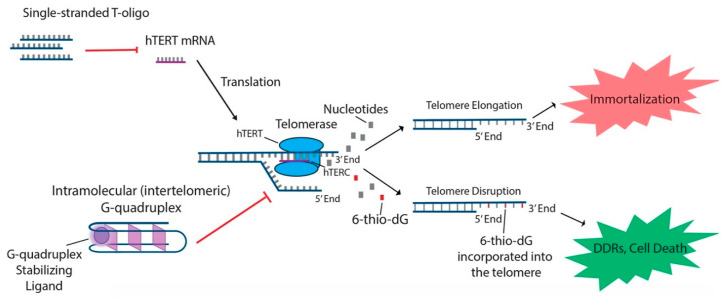
T-oligo, 6-thio-dG, and G-quadruplex stabilizing ligands like telomestatin function to prevent telomere elongation through unique processes related to inhibition of the telomerase subunits. T-oligo has been shown to inhibit expression of hTERT mRNA in melanoma cells with activated JNK signaling. 6-thio-dG incorporates into telomeric DNA and stimulates TIFs in cells that exhibit hTERT activity. G-quadruplex stabilizing ligands prevent hTERC from binding to the single-stranded telomere overhang.

In addition to the aforementioned oligonucleotides, both T-oligos and 6-thio-dG have generated interest due to their potential to regulate hTERT and possible use in treating various cancers. T-oligos are oligonucleotides with a sequence homologous to the 3′ telomere overhang. Their anti-cancer effects are proposed to occur by activation of DNA damage responses (DDRs) and subsequent cell death, either by mimicking damaged DNA or by triggering the dissociation of critical shelterin proteins from telomeres [15,27]. T11 is a specific 11-base T-oligo that has been shown to demonstrate anti-cancer properties in a number of different cancers [8,15,27,28]. T11 is proposed to function through its resemblance to the telomeric overhang DNA sequence and displays promise because it has no effect on normal cells (Figure 2) [27]. Administration of T11 also had no detectable toxic effects in mice [28,95,96,97]. In preclinical studies, T-oligo was shown to inhibit mRNA expression of hTERT by 50% in a melanoma cell model [98]. Additionally, concurrent administration of T-oligo with vemurafenib in V600E-positive melanoma cells demonstrated an additive anti-cancer effect, implicating the potential benefit of T-oligo administration with chemotherapeutics in current use [98,99]. 6-thio-dG, a nucleoside analog, has been shown to possess anti-cancer properties by being incorporated into telomeric DNA, leading to the uncapping of telomeric DNA and dissociation of the shelterin complex (Figure 2). This results in telomere dysfunction-induced foci (TIFs) and subsequent cellular senescence and apoptosis [82]. Furthermore, this effect has been shown to be partly dependent on the activity of hTERT, as TIFs were not seen in cells lacking hTERT activity [82]. Future studies should focus on the specific molecular interactions of 6-thio-dG and hTERT in order to better establish the effect of 6-thio-dG on telomerase activity.

## 4. G-Quadruplex

The human genome contains DNA that is primarily in the duplex state, allowing for the formation of a double helix. However, it is becoming increasingly apparent that non-canonical structures arise in DNA through non-Watson–Crick base pairing [100,101]. One such structure, the G-quadruplex (G4), is formed when a nucleic acid sequence with multiple runs of guanine bases can spontaneously fold into secondary structures. The G4 structure is formed around a tetrad of Hoogsteen hydrogen-bonded guanine residues that are stabilized by monovalent ions including Na^+^ and K^+^ [30]. The tetrads stack on top of each other, forming a multi-layered planar scaffold [26]. In vitro studies have demonstrated that G4 structures are more thermodynamically stable than double-stranded DNA and the kinetics of G4 unfolding are considerably slower than that of double-stranded DNA or RNA hairpin structures [102]. Therefore, since the thermodynamic properties of G4s are likely to interfere with DNA and RNA in transcription, translation, and genomic stability, these structures need some form of regulation [103]. Unlike miRNA and oligonucleotide cancer therapies, which introduce nucleotide structures to target different telomere and telomerase sites, G4 serves as an intramolecular DNA sequence that is inherent to telomere structure, regulates telomerase function, and may be targeted by a diverse range of therapeutics [27,28].

The sequencing of numerous genomes has revealed an abundance of DNA stretches that meet the criteria for potential G4 formation [104]. Interestingly, potential G4 structure sequences are non-randomly distributed within genomes. Approximately 90% of the origins of replication in the human genome contain potential G4 structures [105,106]. Additionally, potential G4 structure sequences have been found to be enriched near the ends of chromosomes, functioning in a regulatory capacity by providing a capping structure for telomeres [30]. Telomeric G4 structures were initially thought to impair telomerase function, but recent studies have suggested that they might also play a role in telomerase recruitment [31,107]. Discovery of telomere repeats containing potential G4 sequences has proposed a link to telomerase-mediated elongation of telomeres, leading researchers to inquire about the use of G4-targeted therapies in cancer treatment through disrupting telomere maintenance.

The process of telomere maintenance has been linked to the function of G4 helicase enzymes [108]. During DNA replication, once the replication fork reaches the telomere sequence, G4 structures must be unwound by helicase enzymes such as BLM, RTEL1, and WRN [26,109,110]. During the cell cycle S phase, WRN has also been shown to localize to telomeres [111]. When WRN does not co-localize to aid in unfolding G4 structures, the replication fork eventually stalls at lagging telomeres and disrupts their replication [112]. The importance of properly unfolding G4 structures during DNA replication highlights the therapeutic potential for G4-stabilizing molecules in the treatment of cancer cells (Figure 2). A few molecules which have demonstrated an ability to disrupt telomere replication and function as anti-cancer agents include BRACO-19, RHPS4, and telomestatin [113,114,115].

Telomestatin functions by stabilizing G4 structures through binding to guanine nucleotides at the 3′ and 5′ ends of telomeres, thus forming a stable complex that inhibits the telomerase RNA component, hTERC, from binding the single stranded overhang [116,117]. Stabilizing G4 structure through telomestatin has resulted in telomerase inhibition in breast, cervical, neuroblastoma, and other cancer cell lines during preclinical studies while also displaying minimal effect on non-cancerous cells [28,32]. However, the drug’s low water solubility resulted in poor bioavailability and has impeded research progress [118]. BRACO-19 and RHPS4 act through a similar mechanism as telomestatin in stabilizing G4 structures, but they have demonstrated less tumor selectivity [119,120].

The repetitive nature of telomere sequences is conducive to many potential G4 structures, providing a target for therapeutic-based G4-forming molecules. T-oligo has demonstrated the ability to form stable intermolecular G4 structures in vitro and induce activation of the SAPK/JNK signaling as well as inhibition of hTERT expression in melanoma cells [25]. Previous studies proposed that JNK activation might result in decreased hTERT expression, thus the anti-cancer effects of T-oligo may be in part due to JNK activation [25,121]. Further interactions with telomeres were highlighted after T-oligo treatment resulted in the upregulation of shelterin complex proteins TRF2 and POT1, offering an additional pathway to telomere dysfunction [25].

The targeting of G4 structures through hTERT promoter mutations has also recently garnered interest. In melanoma, increases in hTERT expression were caused by the same mutations as in 70% of other tumors [29]. Within these tumors, C-to-T sense strand mutations that correspond with G-to-A antisense strand mutations in the promoter of hTERT activated transcription by altering the consensus sequence [122]. Researchers found that these promoter mutations interfered with G4 structure folding by disrupting a silencer element that reduced G4 transcriptional repression [123]. In an attempt to counteract this mutation, a small pharmacological chaperone molecule that acts on the mutated promoter has been proposed as a potential therapeutic [123]. This molecule, GTC365, functioned by partially returning to wild type G4 folding and thus regaining some transcriptional repression ability [123]. In melanoma cells, GTC365 treatment was able to induce cancer cell death through apoptosis and senescence by inhibiting transcription of hTERT and decreasing telomerase activity, ultimately leading to reduced telomere extension [123].

The study of DNA-based G4 structures during the past few decades has progressed from structural analysis to cancer therapy studies in increasingly complex biological systems [124,125,126,127]. Researchers have further uncovered the mechanisms of G4 structures that serve as key factors in a number of DNA and RNA processes. While significant progress has been made in this area, additional studies are needed to better understand G4 function. Some areas of future research should focus on the enrichment of G4 structures near specific cancer genes and the exact mechanism through which these structures affect transcription.

## 5. Conclusions

In this review, we have analyzed the current state of oligonucleotide-based anti-cancer therapeutics that work by targeting telomerase subunits. The mechanistic diversity of this subclass of telomerase inhibitors demonstrates the clinical promise of endogenously derived and exogenously synthesized oligonucleotides for the treatment of various cancers. The administration of miRNAs and AS-ODNs as well as the structural manipulation of telomeric G4s have proven to be effective means of inhibiting hTERT or hTERC, reducing telomerase activity, and slowing tumor growth [24,57,78,79,80,121]. Novel therapies developed in light of these approaches hold the theoretical capacity to target cancer cells expressing telomerase while leaving normal cells unharmed, making them possible alternatives to traditional cancer methods. At the same time, several issues surrounding telomerase inhibition as a potential cancer treatment must be navigated. The long lag time between administration and cancer response, possible deleterious side effects associated with critical telomere shortening, as well as the unknown long-term effects of telomerase inhibition comprise the common criticisms against telomerase inhibition in the treatment of cancer [28,63]. Continued research in the field of oligonucleotide therapies, including how different oligonucleotides interact with each other, telomerase, and telomere architecture, is crucial for the sustained development of innovative cancer treatment regimens.

## Figures and Tables

**Table 1:** A list of important hTERT-targeting tumor suppressor and oncogenic miRNAs, including which cancers each has been studied in and the specific mechanism of action targeting hTERT.

miRNA	Class	Cancers	Action (Targets)
miRNA-1182 [40,41,42]	Tumor Suppressor	Bladder, Gastric	Direct (ORF-1, 3′-UTR)
miRNA-19b [50,51]	Oncogenic	Adenocarcinoma, Breast, Glioma, Lung, Melanoma, Osteosarcoma	Indirect (PITX1)
miRNA-128 [33,48]	Either	Colorectal, Head and Neck, Lung, Prostate, Teratoma,	Direct or Indirect
miRNA-133a [20]	Tumor Suppressor	Adenocarcinoma	Direct (3′-UTR)
miRNA-138 [15,44,45,46,47]	Tumor Suppressor	Thyroid, Cervical, Neuroblastoma	Direct (3′-UTR)
miRNA-21 [15,52,53,54]	Oncogenic	Melanoma, Colorectal, Glioblastoma	Indirect (PTEN, STAT3)
miRNA-342 [20]	Tumor Suppressor	Adenocarcinoma	Direct (3′-UTR)
miRNA-346 [55]	Oncogenic	Cervical	Direct (3′-UTR)
miRNA-491 [20]	Tumor Suppressor	Adenocarcinoma	Direct (3′-UTR)
miRNA-512-5p [43]	Tumor Suppressor	Head and Neck	Direct (3′-UTR)
miRNA-541 [20]	Tumor Suppressor	Adenocarcinoma	Direct (3′-UTR)

## References

[1] Jafri M.A., Ansari S.A., Alqahtani M.H., Shay J.W. (2016). Roles of telomeres and telomerase in cancer, and advances in telomerase-targeted therapies. Genome Med..

[2] Blackburn E.H., Greider C.W., Szostak J.W. (2006). Telomeres and telomerase: The path from maize, Tetrahymena and yeast to human cancer and aging. Nat. Med..

[3] Harley C.B. (2008). Telomerase and cancer therapeutics. Nat. Rev. Cancer.

[4] Tian X., Chen B., Liu X. (2009). Telomere and Telomerase as Targets for Cancer Therapy. Appl. Biochem. Biotechnol..

[5] Giardini M.A., Segatto M., Da Silva M.S., Nunes V.S., Cano M.I.N. (2014). Telomere and Telomerase Biology. Prog. Mol. Biol. Transl. Sci..

[6] Deng Y., Chang S. (2007). Role of telomeres and telomerase in genomic instability, senescence and cancer. Lab. Investig..

[7] Hayflick L., Moorhead P. (1961). The serial cultivation of human diploid cell strains. Exp. Cell Res..

[8] Berei J., Eckburg A., Miliavski E., Anderson A.D., Miller R.J., Dein J., Giuffre A.M., Tang D., Deb S., Racherla K.S. (2020). Potential Telomere-Related Pharmacological Targets. Curr. Top. Med. Chem..

[9] Shammas M.A. (2011). Telomeres, lifestyle, cancer, and aging. Curr. Opin. Clin. Nutr. Metab. Care.

[10] Blackburn E.H. (1992). Telomerases. Annu. Rev. Biochem..

[11] Jiang J., Chan H., Cash D.D., Miracco E.J., Loo R.R.O., Upton H.E., Cascio D., Johnson R.O., Collins K., Loo J.A. (2015). Structure of Tetrahymena telomerase reveals previously unknown subunits, functions, and interactions. Science.

[12] Nguyen T.H.D., Tam J., Wu R.A., Greber B.J., Toso D., Nogales E., Collins K. (2018). Cryo-EM structure of substrate-bound human telomerase holoenzyme. Nature.

[13] Bajaj S., Kumar M.S., Peters G.J., Mayur Y.C. (2020). Targeting telomerase for its advent in cancer therapeutics. Med. Res. Rev..

[14] Kim N.W., A Piatyszek M., Prowse K.R., Harley C.B., West M.D., Ho P.L., Coviello G.M., Wright W.E., Weinrich S.L., Shay J.W. (1994). Specific association of human telomerase activity with immortal cells and cancer. Science.

[15] Schrank Z., Khan N., Osude C., Singh S., Miller R.J., Merrick C., Mabel A., Kuckovic A., Puri N. (2018). Oligonucleotides Targeting Telomeres and Telomerase in Cancer. Molecules.

[16] Shay J., Bacchetti S. (1997). A survey of telomerase activity in human cancer. Eur. J. Cancer.

[17] Shay J.W. (2016). Role of Telomeres and Telomerase in Aging and Cancer. Cancer Discov..

[18] Wright W.E., Shay J.W. (2002). Historical claims and current interpretations of replicative aging. Nat. Biotechnol..

[19] Dean N.M., Bennett C.F. (2003). Antisense oligonucleotide-based therapeutics for cancer. Oncogene.

[20] Hrdlicková R., Nehyba J., Bargmann W., Bose J.H.R. (2014). Multiple Tumor Suppressor microRNAs Regulate Telomerase and TCF7, an Important Transcriptional Regulator of the Wnt Pathway. PLoS ONE.

[21] Stahel R.A., Zangemeister-Wittke U. (2003). Antisense oligonucleotides for cancer therapy—An overview. Lung Cancer.

[22] Zhang B., Pan X., Cobb G.P., Anderson T.A. (2007). microRNAs as oncogenes and tumor suppressors. Dev. Boil..

[23] Leao R., Apolónio J.D., Lee D., Figueiredo A., Tabori U., Castelo-Branco P. (2018). Mechanisms of human telomerase reverse transcriptase (hTERT) regulation: Clinical impacts in cancer. J. Biomed. Sci..

[24] Folini M., Brambilla C., Villa R., Gandellini P., Vignati S., Paduano F., Daidone M.G., Zaffaroni N. (2005). Antisense oligonucleotide-mediated inhibition of hTERT, but not hTERC, induces rapid cell growth decline and apoptosis in the absence of telomere shortening in human prostate cancer cells. Eur. J. Cancer.

[25] Chhabra G., Wojdyla L., Frakes M., Schrank Z., Leviskas B., Ivancich M., Vinay P., Ganapathy R., Ramirez B.E., Puri N. (2018). Mechanism of Action of G-Quadruplex–Forming Oligonucleotide Homologous to the Telomere Overhang in Melanoma. J. Investig. Dermatol..

[26] Crees Z., Girard J., Rios Z., Botting G.M., Harrington K., Shearrow C., Wojdyla L., Stone A.L., Uppada S.B., DeVito J.T. (2014). Oligonucleotides and G-quadruplex stabilizers: Targeting telomeres and telomerase in cancer therapy. Curr. Pharm. Des..

[27] Ivancich M., Schrank Z., Wojdyla L., Leviskas B., Kuckovic A., Sanjali A., Puri N. (2017). Treating Cancer by Targeting Telomeres and Telomerase. Antioxidants.

[28] Ruden M., Puri N. (2013). Novel anticancer therapeutics targeting telomerase. Cancer Treat. Rev..

[29] Horn S., Figl A., Rachakonda P.S., Fischer C., Sucker A., Gast A., Kadel S., Moll I., Nagore E., Hemminki K. (2013). TERT Promoter Mutations in Familial and Sporadic Melanoma. Science.

[30] Rhodes D., Lipps H.J. (2015). G-quadruplexes and their regulatory roles in biology. Nucleic Acids Res..

[31] Zahler A.M., Williamson J.R., Cech T.R., Prescott D.M. (1991). Inhibition of telomerase by G-quartet DMA structures. Nature.

[32] Tahara H., Shin-Ya K., Seimiya H., Yamada H., Tsuruo T., Ide T. (2005). G-Quadruplex stabilization by telomestatin induces TRF2 protein dissociation from telomeres and anaphase bridge formation accompanied by loss of the 3′ telomeric overhang in cancer cells. Oncogene.

[33] Li M.-L., Fu W., Wo L., Shu X., Liu F., Li C.-G. (2013). miR-128 and its target genes in tumorigenesis and metastasis. Exp. Cell Res..

[34] Lund E., Guttinger S., Calado A., Dahlberg J.E., Kutay U. (2004). Nuclear Export of MicroRNA Precursors. Science.

[35] Mikhaĭlova R.I., Bezrukov V.M., Komarova Z.A., Koroleva N.B., Burylina O.M. (1986). [Use of collalysine++ phonophoresis in the combined treatment of hypertrophic and keloid scars of the face and neck]. Stomatologiia (Mosk).

[36] Han J., Lee Y., Yeom K.-H., Kim Y.K., Jin H., Kim V.N. (2004). The Drosha-DGCR8 complex in primary microRNA processing. Genes Dev..

[37] Sarin K.Y., Cheung P., Gilison D., Lee E., Tennen R.I., Wang E., Artandi M.K., Oro A.E., Artandi S.E. (2005). Conditional telomerase induction causes proliferation of hair follicle stem cells. Nature.

[38] Gleason C.E., Cholerton B., Carlsson C.M., Johnson S.C., Asthana S. (2005). Alzheimer’s disease: The impact of age-related changes in reproductive hormones. Cell. Mol. Life Sci..

[39] Esquela-Kerscher A., Slack F.J. (2006). Oncomirs—Micrornas with a role in cancer. Nat. Rev. Cancer.

[40] Zhang D., Xiao Y.F., Zhang J.W., Xie R., Hu C.J., Tang B., Wang S.M., Wu Y.Y., Hao N.B., Yang S.M. (2015). miR-1182 attenuates gastric cancer proliferation and metastasis by targeting the open reading frame of hTERT. Cancer Lett..

[41] Zhou J., Dai W., Song J. (2016). miR-1182 inhibits growth and mediates the chemosensitivity of bladder cancer by targeting hTERT. Biochem. Biophys. Res. Commun..

[42] Hou X.S., Han C.Q., Zhang W. (2018). MiR-1182 inhibited metastasis and proliferation of ovarian cancer by targeting hTERT. Eur. Rev. Med. Pharmacol. Sci..

[43] Li J., Lei H., Xu Y., Tao Z.Z. (2015). miR-512-5p suppresses tumor growth by targeting hTERT in telomerase positive head and neck squamous cell carcinoma in vitro and in vivo. PLoS ONE.

[44] Zhou N., Fei D., Zong S., Zhang M., Yue Y. (2016). MicroRNA-138 inhibits proliferation, migration and invasion through targeting hTERT in cervical cancer. Oncol. Lett..

[45] Mitomo S., Maesawa C., Ogasawara S., Iwaya T., Shibazaki M., Yashima-Abo A., Kotani K., Oikawa H., Sakurai E., Izutsu N. (2008). Downregulation of miR-138 is associated with overexpression of human telomerase reverse transcriptase protein in human anaplastic thyroid carcinoma cell lines. Cancer Sci.

[46] Shukla S., Fu P., Gupta S. (2014). Apigenin induces apoptosis by targeting inhibitor of apoptosis proteins and Ku70-Bax interaction in prostate cancer. Apoptosis.

[47] Chakrabarti M., Banik N.L., Ray S.K. (2013). miR-138 overexpression is more powerful than hTERT knockdown to potentiate apigenin for apoptosis in neuroblastoma in vitro and in vivo. Exp. Cell Res..

[48] Guzman H., Sanders K., Idica A., Bochnakian A., Jury D., Daugaard I., Zisoulis D.G., Pedersen I.M. (2018). miR-128 inhibits telomerase activity by targeting TERT mRNA. Oncotarget.

[49] Shen L., Chen X.D., Zhang Y.H. (2014). MicroRNA-128 promotes proliferation in osteosarcoma cells by downregulating PTEN. Tumour Biol..

[50] Liu D.T., Yao H.R., Li Y.Y., Song Y.Y., Su M.Y. (2018). MicroRNA-19b promotes the migration and invasion of ovarian cancer cells by inhibiting the PTEN/AKT signaling pathway. Oncol. Lett..

[51] Ohira T., Naohiro S., Nakayama Y., Osaki M., Okada F., Oshimura M., Kugoh H. (2015). miR-19b regulates hTERT mRNA expression through targeting PITX1 mRNA in melanoma cells. Sci. Rep..

[52] Melnik B.C. (2015). MiR-21: An environmental driver of malignant melanoma?. J. Transl. Med..

[53] Yang Y., Yang J.J., Tao H., Jin W.S. (2015). MicroRNA-21 controls hTERT via PTEN in human colorectal cancer cell proliferation. J. Physiol. Biochem..

[54] Zhu H.Y., Li C., Bai W.D., Su L.L., Liu J.Q., Li Y., Shi J.H., Cai W.X., Bai X.Z., Jia Y.H. (2014). MicroRNA-21 regulates hTERT via PTEN in hypertrophic scar fibroblasts. PLoS ONE.

[55] Song G., Wang R., Guo J., Liu X., Wang F., Qi Y., Wan H., Liu M., Li X., Tang H. (2015). miR-346 and miR-138 competitively regulate hTERT in GRSF1- and AGO2-dependent manners, respectively. Sci. Rep..

[56] Gandhi N.S., Tekade R.K., Chougule M.B. (2014). Nanocarrier mediated delivery of siRNA/miRNA in combination with chemotherapeutic agents for cancer therapy: Current progress and advances. J. Control Release.

[57] Nguyen D.D., Chang S. (2017). Development of Novel Therapeutic Agents by Inhibition of Oncogenic MicroRNAs. Int. J. Mol. Sci..

[58] Wang Y.Y., Sun G., Luo H., Wang X.F., Lan F.M., Yue X., Fu L.S., Pu P.Y., Kang C.S., Liu N. (2012). MiR-21 modulates hTERT through a STAT3-dependent manner on glioblastoma cell growth. CNS Neurosci. Ther..

[59] Zhuang C.L., Fu X., Liu L., Liu Y.C., Huang W.R., Cai Z.M. (2015). Synthetic miRNA sponges driven by mutant hTERT promoter selectively inhibit the progression of bladder cancer. Tumour Biol..

[60] Thomas J.R., Hergenrother P.J. (2008). Targeting RNA with Small Molecules. Chem. Rev..

[61] Chang H., Yi B., Ma R., Zhang X., Zhao H., Xi Y. (2016). CRISPR/cas9, a novel genomic tool to knock down microRNA in vitro and in vivo. Sci. Rep..

[62] Huo W., Zhao G., Yin J., Ouyang X., Wang Y., Yang C., Wang B., Dong P., Wang Z., Watari H. (2017). Lentiviral CRISPR/Cas9 vector mediated miR-21 gene editing inhibits the epithelial to mesenchymal transition in ovarian cancer cells. J. Cancer.

[63] Burchett K.M., Yan Y., Ouellette M.M. (2014). Telomerase Inhibitor Imetelstat (GRN163L) Limits the Lifespan of Human Pancreatic Cancer Cells. PLoS ONE.

[64] Jäger K., Walter M. (2016). Therapeutic Targeting of Telomerase. Genes.

[65] Guo Z., Maki M., Ding R., Yang Y., Zhang B., Xiong L. (2014). Genome-wide survey of tissue-specific microRNA and transcription factor regulatory networks in 12 tissues. Sci. Rep..

[66] Frixa T., Donzelli S., Blandino G. (2015). Oncogenic MicroRNAs: Key Players in Malignant Transformation. Cancers.

[67] Bertorelle R., Briarava M., Rampazzo E., Biasini L., Agostini M., Maretto I., Lonardi S., Friso M.L., Mescoli C., Zagonel V. (2013). Telomerase is an independent prognostic marker of overall survival in patients with colorectal cancer. Br. J. Cancer.

[68] Bianchi F., Nicassio F., Marzi M., Belloni E., Dall’Olio V., Bernard L., Pelosi G., Maisonneuve P., Veronesi G., Di Fiore P.P. (2011). A serum circulating miRNA diagnostic test to identify asymptomatic high-risk individuals with early stage lung cancer. EMBO Mol. Med..

[69] Moltzahn F., Olshen A.B., Baehner L., Peek A., Fong L., Stöppler H., Simko J., Hilton J.F., Carroll P., Blelloch R. (2010). Microfluidic-based multiplex qRT-PCR identifies diagnostic and prognostic microRNA signatures in the sera of prostate cancer patients. Cancer Res..

[70] Deblakshmi R.K., Deka M., Saikia A.K., Sharma B.K., Singh N., Das N.N., Bose S. (2015). Prognostic Relevance of Human Telomerase Reverse Transcriptase (hTERT) Expression in Patients with Gall Bladder Disease and Carcinoma. Asian Pac. J. Cancer Prev..

[71] Alm E.W., Oerther D.B., Larsen N., A Stahl D., Raskin L. (1996). The oligonucleotide probe database. Appl. Environ. Microbiol..

[72] Dalbadie-McFarland G., Cohen L.W., Riggs A.D., Morin C., Itakura K., Richards J.H. (1982). Oligonucleotide-directed mutagenesis as a general and powerful method for studies of protein function. Proc. Natl. Acad. Sci. USA.

[73] Corey D.R. (2002). Telomerase inhibition, oligonucleotides, and clinical trials. Oncogene.

[74] Cimino-Reale G., Gandellini P., Santambrogio F., Recagni M., Zaffaroni N., Folini M. (2017). miR-380-5p-mediated repression of TEP1 and TSPYL5 interferes with telomerase activity and favours the emergence of an “ALT-like” phenotype in diffuse malignant peritoneal mesothelioma cells. J. Hematol. Oncol..

[75] Hannen R., Bartsch J.W. (2018). Essential roles of telomerase reverse transcriptase hTERT in cancer stemness and metastasis. FEBS Lett..

[76] Lü M.-H., Liao Z.-L., Zhao X.-Y., Fan Y.-H., Lin X.-L., Fang D.-C., Guo H., Yang S.-M. (2012). hTERT-based therapy: A universal anticancer approach (Review). Oncol. Rep..

[77] Satyanarayana A., Manns M.P., Rudolph K.L. (2004). Telomeres, telomerase and cancer: An endless search to target the ends. Cell Cycle.

[78] Yang B., Yu R.-L., Tuo S., Tuo C.-W., Liu Q.-Z., Zhang N., Lu X.-C., Chi X.-H., Lv S.-B., Cai L.-L. (2012). Antisense Oligonucleotide against hTERT (Cantide) Inhibits Tumor Growth in an Orthotopic Primary Hepatic Lymphoma Mouse Model. PLoS ONE.

[79] Liu S.-X., Sun W.-S., Cao Y.-L., Ma C.-H., Han L.-H., Zhang L.-N., Wang Z.-G., Zhu F.-L. (2004). Antisense oligonucleotide targeting at the initiator of hTERT arrests growth of hepatoma cells. World J. Gastroenterol..

[80] Kraemer K., Fuessel S., Schmidt U., Kotzsch M., Schwenzer B., Wirth M., Meye A. (2003). Antisense-mediated hTERT inhibition specifically reduces the growth of human bladder cancer cells. Clin. Cancer Res..

[81] Fu X.-H., Zhang J.-S., Zhang N., Zhang Y.-D. (2005). Combination of telomerase antisense oligonucleotides simultaneously targeting hTR and hTERT produces synergism of inhibition of telomerase activity and growth in human colon cancer cell line. World J. Gastroenterol..

[82] Mender I., Gryaznov S., Dikmen Z.G., Wright W.E., Shay J.W. (2014). Induction of telomere dysfunction mediated by the telomerase substrate precursor 6-thio-2′-deoxyguanosine. Cancer Discov..

[83] Lin R.-X., Tuo C.-W., Lü Q.-J., Zhang W., Wang S.-Q. (2005). Inhibition of tumor growth and metastasis with antisense oligonucleotides (Cantide) targeting hTERT in an in situ human hepatocellular carcinoma model. Acta Pharmacol. Sin..

[84] Frink R.E., Peyton M., Schiller J.H., Gazdar A.F., Shay J.W., Minna J.D. (2016). Telomerase inhibitor imetelstat has preclinical activity across the spectrum of non-small cell lung cancer oncogenotypes in a telomere length dependent manner. Oncotarget.

[85] Sugarman E.T., Zhang G., Shay J.W. (2019). In perspective: An update on telomere targeting in cancer. Mol. Carcinog..

[86] A Shammas M., Koley H., Bertheau R.C., Neri P., Fulciniti M., Tassone P., Blotta S., Protopopov A., Mitsiades C., Batchu R.B. (2008). Telomerase inhibitor GRN163L inhibits myeloma cell growth in vitro and in vivo. Leukemia.

[87] Hu Y., Bobb D., Lu Y., He J., Dome J.S. (2014). Effect of telomerase inhibition on preclinical models of malignant rhabdoid tumor. Cancer Genet..

[88] Mender I., Senturk S., Ozgunes N., Akcali K.C., Kletsas D., Gryaznov S., Can A., Shay J.W., Dikmen Z.G. (2013). Imetelstat (a telomerase antagonist) exerts off-target effects on the cytoskeleton. Int. J. Oncol..

[89] Dikmen Z.G., Gellert G.C., Jackson S., Gryaznov S., Tressler R., Dogan P., Wright W.E., Shay J.W. (2005). In vivoInhibition of Lung Cancer by GRN163L: A Novel Human Telomerase Inhibitor. Cancer Res..

[90] Kubasch A.S., Platzbecker U. (2019). Setting Fire to ESA and EMA Resistance: New Targeted Treatment Options in Lower Risk Myelodysplastic Syndromes. Int. J. Mol. Sci..

[91] Salloum R., Hummel T.R., Kumar S.S., Dorris K., Li S., Lin T., Daryani V.M., Stewart C.F., Miles L., Poussaint T.Y. (2016). A molecular biology and phase II study of imetelstat (GRN163L) in children with recurrent or refractory central nervous system malignancies: A pediatric brain tumor consortium study. J. Neuro-Oncology.

[92] Chiappori A., Kolevska T., Spigel D.R., Hager S., Rarick M., Gadgeel S., Blais N., Von Pawel J., Hart L., Reck M. (2015). A randomized phase II study of the telomerase inhibitor imetelstat as maintenance therapy for advanced non-small-cell lung cancer. Ann. Oncol..

[93] Cong Y., Shay J.W. (2008). Actions of human telomerase beyond telomeres. Cell Res..

[94] Uziel O., Beery E., Dronichev V., Samocha K., Gryaznov S., Weiss L., Slavin S., Kushnir M., Nordenberg Y., Rabinowitz C. (2010). Telomere Shortening Sensitizes Cancer Cells to Selected Cytotoxic Agents: In Vitro and In Vivo Studies and Putative Mechanisms. PLoS ONE.

[95] Eller M.S., Puri N., Hadshiew I.M., Venna S.S., Gilchrest B.A. (2002). Induction of Apoptosis by Telomere 3′ Overhang-Specific DNA. Exp. Cell Res..

[96] Puri N., Eller M.S., Byers H.R., Dykstra S., Kubera J., Gilchrest B.A. (2004). Telomere-based DNA damage responses: A new approach to melanoma. FASEB J..

[97] Puri N., Pitman R.T., Mulnix R.E., Erickson T., Iness A.N., Vitali C., Zhao Y., Salgia R. (2013). Non-small cell lung cancer is susceptible to induction of DNA damage responses and inhibition of angiogenesis by telomere overhang oligonucleotides. Cancer Lett..

[98] Khan N., Schrank Z., Kellen J., Singh S., Osude C., Puri N., Chhabra G. (2018). Abstract 1469: T-oligo mediates DNA damage responses by modulating telomere associated proteins and telomerase. Am. Assoc. Cancer Res..

[99] Schrank Z., Khan N., Kellen J., Singh S., Osude C., Puri N. (2019). Abstract 2965: Induction of DNA damage responses by T-oligo and 6-thio-dG via modulating telomere associated proteins and telomerase. Am. Assoc. Cancer Res..

[100] Lipps H.J., Rhodes D. (2009). G-quadruplex structures: In vivo evidence and function. Trends Cell Boil..

[101] Mukherjee A.K., Sharma S., Chowdhury S. (2018). Non-duplex G-Quadruplex Structures Emerge as Mediators of Epigenetic Modifications. Trends Genet..

[102] Lane A.N., Chaires J.B., Gray R.D., Trent J.O. (2008). Stability and kinetics of G-quadruplex structures. Nucleic Acids Res..

[103] Varshney D., Spiegel J., Zyner K., Tannahill D., Balasubramanian S. (2020). The regulation and functions of DNA and RNA G-quadruplexes. Nat. Rev. Mol. Cell Boil..

[104] Huppert J.L., Balasubramanian S. (2005). Prevalence of quadruplexes in the human genome. Nucleic Acids Res..

[105] Cayrou C., Gregoire D., Coulombe P., Danis E., Méchali M. (2012). Genome-scale identification of active DNA replication origins. Methods.

[106] Besnard E., Babled A., Lapasset L., Milhavet O., Parrinello H., Dantec C., Marin J.-M., Lemaitre J.-M. (2012). Unraveling cell type–specific and reprogrammable human replication origin signatures associated with G-quadruplex consensus motifs. Nat. Struct. Mol. Boil..

[107] Moye A.L., Porter K.C., Cohen S.B., Phan T., Zyner K.G., Sasaki N., Lovrecz G.O., Beck J.L., Bryan T. (2015). Telomeric G-quadruplexes are a substrate and site of localization for human telomerase. Nat. Commun..

[108] Brosh R.M. (2013). DNA helicases involved in DNA repair and their roles in cancer. Nat. Rev. Cancer.

[109] Fry M., Loeb L.A. (1999). Human werner syndrome DNA helicase unwinds tetrahelical structures of the fragile X syndrome repeat sequence d(CGG)n. J. Boil. Chem..

[110] Damerla R., Knickelbein K.E., Strutt S., Liu F.-J., Wang H., Opresko P.L. (2012). Werner syndrome protein suppresses the formation of large deletions during the replication of human telomeric sequences. Cell Cycle.

[111] Crabbe L., Verdun R.E., Haggblom C.I., Karlseder J. (2004). Defective Telomere Lagging Strand Synthesis in Cells Lacking WRN Helicase Activity. Science.

[112] Arnoult N., Saintomé C., Ourliac-Garnier I., Riou J.F., Londoño-Vallejo A. (2009). Human POT1 is required for efficient telomere C-rich strand replication in the absence of WRN. Genes Dev..

[113] Burger A.M. (2005). The G-Quadruplex-Interactive Molecule BRACO-19 Inhibits Tumor Growth, Consistent with Telomere Targeting and Interference with Telomerase Function. Cancer Res..

[114] Tauchi T., Shin-Ya K., Sashida G., Sumi M., Okabe S., Ohyashiki J.H., Ohyashiki K. (2006). Telomerase inhibition with a novel G-quadruplex-interactive agent, telomestatin: In vitro and in vivo studies in acute leukemia. Oncogene.

[115] Salvati E., Leonetti C., Rizzo A., Scarsella M., Mottolese M., Galati R., Sperduti I., Stevens M.F., D’Incalci M., Blasco M.A. (2007). Telomere damage induced by the G-quadruplex ligand RHPS4 has an antitumor effect. J. Clin. Investig..

[116] Monchaud D., Granzhan A., Saettel N., Guédin A., Mergny J.-L., Teulade-Fichou M.-P. (2010). “One Ring to Bind Them All”—Part I: The Efficiency of the Macrocyclic Scaffold for G-Quadruplex DNA Recognition. J. Nucleic Acids.

[117] Gomez D.M., Armando R., Cerrudo C., Ghiringhelli P.D., Gómez D.E. (2016). Telomerase as a Cancer Target. Development of New Molecules. Curr. Top. Med. Chem..

[118] Sullivan H.-J., Readmond C., Radicella C., Persad V., Fasano T.J., Wu C. (2018). Binding of Telomestatin, TMPyP4, BSU6037, and BRACO19 to a Telomeric G-Quadruplex–Duplex Hybrid Probed by All-Atom Molecular Dynamics Simulations with Explicit Solvent. ACS Omega.

[119] Taetz S., Baldes C., Mürdter T.E., Kleideiter E., Piotrowska K., Bock U., Haltner-Ukomadu E., Mueller J., Huwer H., Schaefer U.F. (2006). Biopharmaceutical Characterization of the Telomerase Inhibitor BRACO19. Pharm. Res..

[120] Gowan S.M., Harrison J.R., Patterson L., Valenti M., Read M.A., Neidle S., Kelland L.R. (2002). A G-quadruplex-interactive potent small-molecule inhibitor of telomerase exhibiting in vitro and in vivo antitumor activity. Mol. Pharmacol..

[121] Tian X., Dai S., Sun J., Jiang S., Sui C., Meng F., Li Y., Fu L., Jiang T., Wang Y. (2015). Bufalin Induces Mitochondria-Dependent Apoptosis in Pancreatic and Oral Cancer Cells by Downregulating hTERT Expression via Activation of the JNK/p38 Pathway. Evid.-Based Complement Altern. Med..

[122] Huang F.W., Hodis E., Xu M.J., Kryukov G.V., Chin L., Garraway L.A. (2013). Highly Recurrent TERT Promoter Mutations in Human Melanoma. Science.

[123] Kang H.-J., Cui Y., Yin H., Scheid A., Hendricks W.P., Schmidt J., Sekulic A., Kong D., Trent J.M., Gokhale V. (2016). A Pharmacological Chaperone Molecule Induces Cancer Cell Death by Restoring Tertiary DNA Structures in Mutant hTERT Promoters. J. Am. Chem. Soc..

[124] Carvalho J., Mergny J.-L., Salgado G.F., Queiroz J.A., Cruz C. (2020). G-quadruplex, Friend or Foe: The Role of the G-quartet in Anticancer Strategies. Trends Mol. Med..

[125] Gellert M., Lipsett M.N., Davies D.R. (1962). Helix formation by guanylic acid. Proc. Natl. Acad. Sci. USA.

[126] Kendrick S., Muranyi A., Gokhale V., Hurley L.H., Rimsza L.M. (2017). Simultaneous Drug Targeting of the Promoter MYC G-Quadruplex and BCL2 i-Motif in Diffuse Large B-Cell Lymphoma Delays Tumor Growth. J. Med. Chem..

[127] Rodriguez R., Miller K.M., Forment J.V., Bradshaw C.R., Nikan M., Britton S., Oelschlaegel T., Xhemalçe B., Balasubramanian S., Jackson S.P. (2012). Small-molecule–induced DNA damage identifies alternative DNA structures in human genes. Nat. Methods.

